# The Consequences of Prenatal Tobacco Exposure: A Systematic Review of Birth Outcomes and Developmental Trajectories

**DOI:** 10.7759/cureus.105888

**Published:** 2026-03-26

**Authors:** Kadambari Ambildhok, Kailash Asawa

**Affiliations:** 1 Department of Dentistry, Pacific Academy of Higher Education and Research, Udaipur, IND

**Keywords:** low birth weight, maternal smoking, neurodevelopment, prenatal tobacco exposure, systematic review

## Abstract

Prenatal tobacco exposure remains a significant and preventable public health concern worldwide. Maternal smoking during pregnancy has been associated with adverse perinatal outcomes and long-term developmental consequences. This systematic review aimed to evaluate the association between prenatal tobacco exposure and birth and developmental outcomes in offspring.

A systematic review was conducted in accordance with PRISMA 2020 guidelines. Six electronic databases were searched for observational studies published between June 1, 2014, and June 1, 2025. Studies assessing maternal tobacco exposure during pregnancy and reporting quantitative associations with birth or developmental outcomes were included. Risk of bias was assessed using the ROBINS-I tool, and the certainty of evidence was evaluated using the GRADE approach.

Twelve studies met the inclusion criteria. Prenatal tobacco exposure was consistently associated with increased risk of low birth weight, with adjusted odds ratios ranging from 2.24 to 2.91. Reported reductions in birth weight ranged from approximately 230 g to over 300 g among exposed infants. Biomarker-validated studies confirmed reductions in birth weight and head circumference. Developmental outcomes demonstrated impairments in attention, executive function, fine motor skills, and academic performance. Increased risks of psychiatric disorders, including attention-deficit/hyperactivity disorder and disruptive behavior disorder, were observed in longitudinal cohorts. Most studies were rated as having a moderate risk of bias. Certainty of evidence was graded as moderate for low birth weight and neurodevelopmental outcomes, and low for psychiatric outcomes and smokeless tobacco exposure.

Prenatal tobacco exposure is consistently associated with reduced fetal growth and adverse neurodevelopmental and psychiatric outcomes extending into later life. Evidence regarding smokeless tobacco exposure remains limited. These findings support the continued prioritization of smoking cessation interventions during pregnancy to reduce preventable adverse child health outcomes.

## Introduction and background

Tobacco use during pregnancy remains a significant and preventable global public health concern. Estimates suggest that approximately 8%-15% of pregnant women continue to smoke despite established clinical guidelines and public health warnings [1,2]. Nicotine readily crosses the placental barrier and may disrupt fetal growth and neurodevelopment through direct toxic and vasoconstrictive effects [3].

Globally, tobacco use during pregnancy varies considerably by region, with higher prevalence reported in certain high-income countries and persistent use in low- and middle-income settings despite public health interventions [2,4]. The World Health Organization estimates that millions of infants are exposed to tobacco in utero annually, contributing substantially to preventable perinatal morbidity and mortality [1]. In addition, socioeconomic disparities strongly influence smoking persistence during pregnancy, with higher prevalence observed among women with lower educational attainment and limited access to cessation resources [2].

Extensive epidemiological evidence has consistently linked prenatal tobacco exposure (PTE) to adverse perinatal outcomes, including low birth weight (LBW), preterm birth (PTB), and small for gestational age (SGA) status [4,5]. These early-life complications are clinically important not only because of immediate neonatal morbidity but also due to their association with longer-term cognitive, behavioral, and respiratory consequences [6]. Additionally, placental dysfunction, impaired oxygen transport, and reduced nutrient exchange have been implicated as biological mechanisms underlying these associations [7,8]. Beyond placental vascular compromise, nicotine interacts with nicotinic acetylcholine receptors in the developing fetal brain, potentially altering neuronal proliferation, differentiation, and synaptic maturation [3]. Experimental models have demonstrated long-term alterations in dopaminergic and serotonergic signaling pathways following prenatal nicotine exposure, providing mechanistic support for later behavioral dysregulation [3].

In addition to nicotine, carbon monoxide (CO) plays a critical role in mediating fetal risk associated with maternal smoking. Carbon monoxide binds to maternal and fetal hemoglobin with an affinity approximately 200-250 times greater than that of oxygen, forming carboxyhemoglobin and significantly reducing oxygen-carrying capacity. This results in chronic fetal hypoxia and impaired oxygen delivery to rapidly developing tissues. Furthermore, carbon monoxide exposure has been associated with placental vascular dysfunction, reduced uteroplacental blood flow, and impaired nutrient exchange, contributing to intrauterine growth restriction and low birth weight [2,4]. Persistent hypoxic stress during critical periods of organogenesis may also disrupt normal brain development, thereby increasing susceptibility to later neurodevelopmental and psychiatric outcomes. These mechanistic pathways provide biological plausibility for the consistent epidemiological associations observed between prenatal tobacco exposure and adverse fetal and long-term developmental outcomes [3,9].

Emerging research suggests that the impact of prenatal tobacco exposure may extend beyond structural growth effects to include epigenetic alterations. Genome-wide studies have demonstrated differential DNA methylation patterns in newborns exposed to maternal smoking during pregnancy, with some changes persisting across the life course [7,10]. These findings provide mechanistic support for observed associations between in utero exposure and later neurodevelopmental and psychiatric outcomes.

Although the harmful effects of active smoking during pregnancy are well established, environmental tobacco smoke and smokeless tobacco use have also been associated with adverse pregnancy outcomes in certain populations [11,12]. Furthermore, variations in exposure assessment (self-report versus biomarker validation), outcome measurement, and control for confounding factors contribute to heterogeneity in findings [2,5].

Previous systematic reviews and meta-analyses have primarily focused on perinatal outcomes, such as low birth weight and preterm birth, associated with maternal smoking during pregnancy [4,13,14]. While these studies have established a strong association between prenatal tobacco exposure and adverse birth outcomes, relatively few reviews have comprehensively examined long-term developmental, neurocognitive, and psychiatric trajectories. Additionally, emerging evidence from biomarker-based studies and epigenetic research has not been fully integrated into earlier syntheses. Therefore, the present systematic review aims to provide an updated and comprehensive evaluation by integrating both immediate birth outcomes and long-term developmental consequences associated with prenatal tobacco exposure.

Although numerous studies have examined the impact of prenatal tobacco exposure on isolated birth or developmental outcomes, variability in exposure measurement, outcome assessment, and adjustment for confounding factors has contributed to heterogeneous findings. Moreover, increasing evidence regarding epigenetic modifications and long-term psychiatric trajectories warrants an updated synthesis. Therefore, a comprehensive systematic review integrating both immediate birth outcomes and long-term developmental trajectories is needed to clarify the magnitude, consistency, and biological plausibility of these associations.

## Review

Materials and methods

Study Design and Registration

This systematic review was conducted to evaluate the association between prenatal tobacco exposure (PTE) and adverse birth and developmental outcomes in offspring. The review was conducted and reported in accordance with PRISMA 2020 guidelines, ensuring transparency in study selection, data extraction, and reporting [9]. The study protocol was prospectively registered with the International Prospective Register of Systematic Reviews (PROSPERO) under registration number CRD42024555533 to ensure methodological transparency and reduce the risk of selective reporting. The literature search included studies published between June 1, 2014, and June 1, 2025. A total of 536 records were identified, and after screening and eligibility assessment, 12 studies met the inclusion criteria and were included in the qualitative synthesis.

Eligibility Criteria

Eligibility criteria were defined using the PECO framework. The population included pregnant women and their offspring, irrespective of geographic region, socioeconomic background, or healthcare setting. The exposure of interest was maternal tobacco use during pregnancy, including active cigarette smoking and smokeless tobacco products. The comparator group consisted of pregnant women with no reported tobacco exposure during pregnancy. Primary outcomes included low birth weight (LBW), preterm birth (PTB), small for gestational age (SGA), stillbirth, and miscarriage. Secondary outcomes comprised developmental and long-term health outcomes in offspring, including cognitive impairment, language delay, behavioral disorders such as attention-deficit/hyperactivity disorder, motor delay, emotional disturbances, respiratory morbidity, growth abnormalities, and chronic conditions. Only primary observational studies (cohort, case-control, and cross-sectional designs) reporting quantitative associations between prenatal tobacco exposure and at least one specified outcome were included. Randomized controlled trials that deliberately assign pregnant women to tobacco exposure would be unethical and therefore were not expected. Although randomized trials may exist for related interventions, such as smoking cessation during pregnancy, they are not appropriate for evaluating the harmful effects of prenatal tobacco exposure itself. Accordingly, observational studies were considered the most appropriate and strongest available design for assessing associations between prenatal tobacco exposure and offspring outcomes.

Information Sources and Search Strategy

A comprehensive literature search was conducted across six electronic databases: PubMed, Scopus, ScienceDirect, Google Scholar, Cochrane Library, and Dimensions. The search included studies published between June 1, 2014 and June 1, 2025. A combination of Medical Subject Headings (MeSH) terms and free-text keywords was used. The complete search strategy is provided in Supplementary Material (see Appendices). Duplicate records were removed using reference management software.

Study Selection

**Figure 1:**
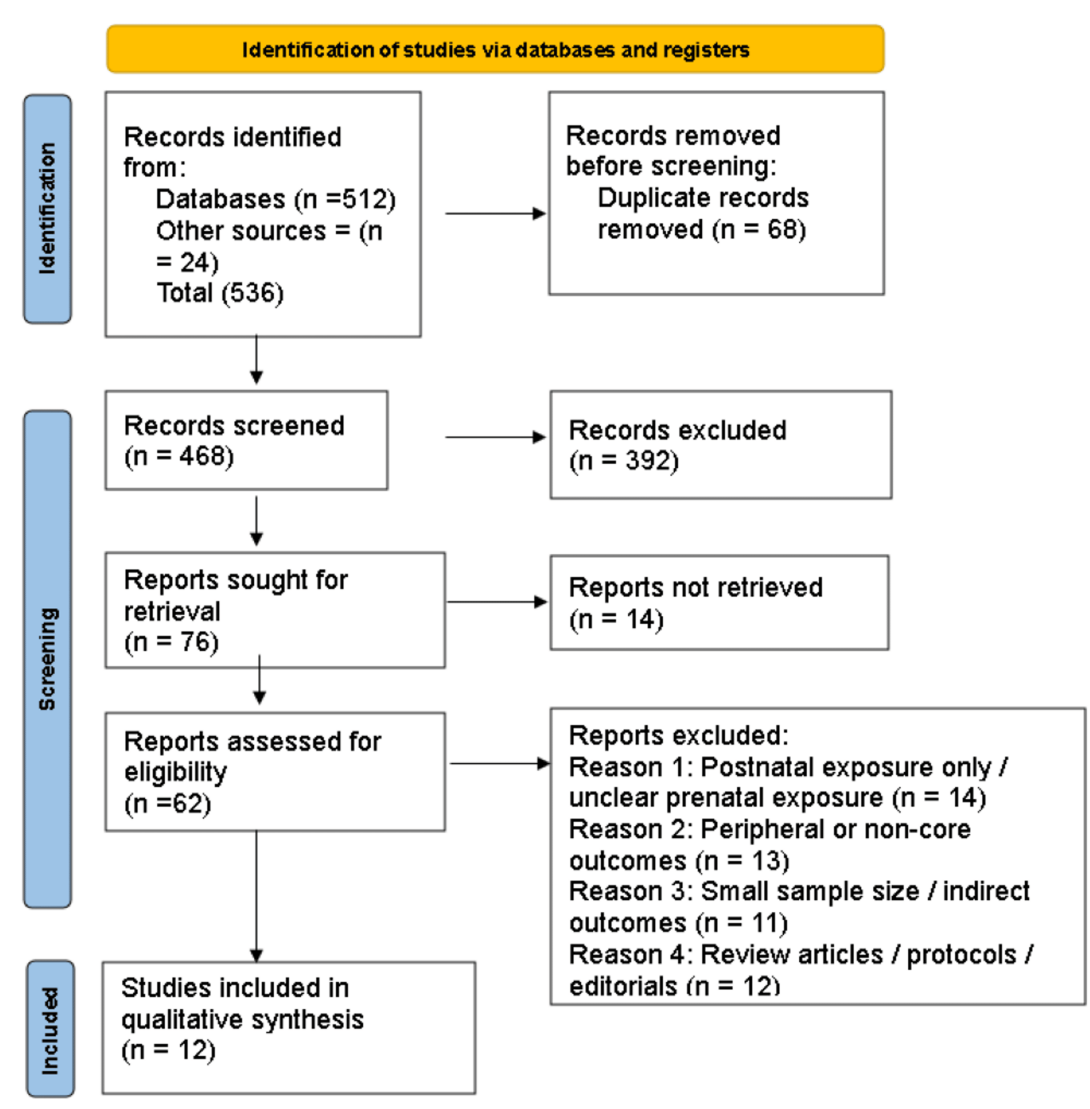
PRISMA flowchart

Two reviewers independently screened titles and abstracts of identified records. Full-text articles were retrieved for potentially eligible studies. Disagreements were resolved through consensus discussion. The study selection process is illustrated in Figure 1 (PRISMA 2020 flow diagram).

Data Extraction

Data extraction was performed using a standardized and pre-piloted Microsoft Excel form. Extracted variables included study characteristics (author, year, country, study design, sample size, follow-up duration), exposure details (type, timing, dose, assessment method), outcome measures (birth and developmental outcomes), and statistical metrics (adjusted effect estimates, confidence intervals, p-values, and covariates).

Data extraction was conducted independently by two reviewers, and discrepancies were resolved by consensus. Data extraction was performed using Microsoft Excel (Microsoft Corporation, Redmond, WA, USA; Version 365, 2023), and risk of bias tables and evidence synthesis were prepared using Review Manager Web (RevMan Web, The Cochrane Collaboration, 2024).

Risk of Bias Assessment

The methodological quality of included studies was assessed using the Risk Of Bias In Non-randomized Studies of Interventions (ROBINS-I) tool [10]. Bias was evaluated across seven domains. The overall risk of bias for each study was determined by the highest level of risk identified across domains.

Meta-Analysis

A meta-analysis was not performed due to substantial clinical and methodological heterogeneity across the included studies. Variations were observed in exposure definitions (active smoking, secondhand smoke, and smokeless tobacco), outcome measurements (categorical versus continuous birth weight and diverse developmental outcomes), and reported effect estimates (odds ratios, relative risks, and beta coefficients). Additionally, differences in study design, population characteristics, and adjustment for confounding factors limited comparability across studies. Therefore, a narrative synthesis was considered more appropriate.

Certainty of Evidence

The certainty of evidence for each outcome domain was assessed using the Grading of Recommendations Assessment, Development and Evaluation (GRADE) framework.

Results

Study Selection

A total of 536 records were identified through database and manual searches. After the removal of 68 duplicates, 468 records were screened. Of these, 392 were excluded based on title and abstract screening. Seventy-six full-text articles were sought for retrieval, and 62 were assessed for eligibility after exclusion of 14 reports that could not be retrieved. Fifty studies were excluded for reasons including postnatal-only exposure, peripheral or non-core outcomes, small sample size, or inappropriate study design. Twelve studies met the inclusion criteria and were included in the qualitative synthesis.

Study Characteristics

The 12 included studies were conducted across Asia, Europe, Africa, and North America. Study designs included case-control studies, longitudinal cohort studies, registry-based cohorts, cross-sectional analyses, and one secondary analysis of a randomized trial. Sample sizes ranged from 150 to over 73,000 participants. Most studies evaluated maternal cigarette smoking during pregnancy, whereas one study specifically examined smokeless tobacco use [12]. Outcomes assessed included birth weight, gestational age, head circumference, neurodevelopmental performance, academic achievement, and long-term psychiatric outcomes. Detailed characteristics of included studies are presented in Table 1.

**Table 1:** Study characteristics of included studies LBW: low birth weight; SLT: smokeless tobacco; ADHD: attention-deficit/hyperactivity disorder; PTB: preterm birth; RCT; randomized controlled trial; LMIC: low and middle-income countries

No.	Author (year)	Country	Study design	Key outcome domain	Rationale for inclusion
1	Agena et al. (2020) [11]	Ethiopia	Case-control	LBW	Strong association in LMIC setting
2	Aziz Ali et al. (2021) [12]	Pakistan	Secondary RCT analysis	SLT, LBW	Key smokeless tobacco study
3	Ashford et al. (2021) [13]	USA	Longitudinal	Birth weight, gestational age	Cessation effects, biomarker validation
4	Browne et al. (2016) [14]	Denmark	Cohort	Psychiatric outcomes	Large population-based cohort
5	Chelchowska et al. (2016) [15]	Poland	Comparative	Birth weight, head circumference	Placental biomarkers
6	Ein-Mor et al. (2019) [16]	Israel	Cohort	Birth weight, head circumference	Cotinine-validated exposure
7	Fuemmeler et al. (2023) [17]	USA	Longitudinal	Cognition, ADHD	Multi-domain neurodevelopment
8	Kristjansson et al. (2018) [18]	Iceland	Registry cohort	Academic outcomes	Long-term trajectories
9	Moore et al. (2019) [19]	USA	Cohort	Motor, executive function	Strong effect sizes
10	Rozi et al. (2016) [20]	Pakistan	Case-control	LBW, PTB	Strong regional evidence
11	Talati et al. (2017) [21]	USA	Longitudinal	Adult psychiatric outcomes	Life-course outcomes
12	Wang et al. (2024) [22]	China	Cross-sectional	LBW, secondhand smoke	Large population study

Birth Outcomes

Most studies demonstrated a significant association between prenatal tobacco exposure and adverse birth outcomes. Maternal smoking during pregnancy was consistently associated with increased risk of low birth weight (LBW) and reduced fetal growth (Table 2).

**Table 2:** Birth outcomes associated with prenatal tobacco exposure LBW: low birth weight; PTB: preterm birth; BW: birth weight; GA: gestational age; AOR: adjusted odds ratio; OR: odds ratio; RR: relative risk; CI: confidence interval; NS: not significant; BMI: body mass index; ANC: antenatal care; SES: socioeconomic status

Study	Exposure timing	Outcome(s)	Measurement	Effect estimate	Statistical significance	Adjusted confounders
Agena et al. [11]	Prenatal	LBW	Interview, anthropometry	AOR=2.31	p<0.05	Age, ANC, iron intake
Aziz Ali et al. [12]	Pre-conception	LBW, PTB	Registry	RR=0.96	NS	Age, BMI, income
Ashford et al. [13]	Each trimester	BW, GA	Medical records	Higher BW in quitters	p<0.05	Baseline smoking
Browne et al. [14]	Throughout	Psychiatric outcomes	National registry	aHR=1.66	95% CI: 1.17-2.35	Psychiatric history
Chelchowska et al. [15]	All trimesters	BW, HC	PAPP-A	β=-230 g	p<0.001	GA, trimester
Ein-Mor et al. [16]	Perinatal	BW, HC	Urinary cotinine	Reduced BW	p<0.05	Maternal weight
Rozi et al. [20]	During pregnancy	LBW, PTB	Records	LBW OR=2.24	p<0.05	Age, parity
Talati et al. [21]	Daily smoking	BW	Birth records	-317 g	p=0.0002	SES, psychiatric history
Wang et al. [22]	Prenatal	LBW	Registry	OR=2.91	95% CI: 1.49-5.68	BMI, income

Agena et al. [11] reported more than a twofold increase in LBW risk among exposed infants (adjusted odds ratio [AOR] = 2.31). Wang et al. [22] observed increased odds of LBW associated with both firsthand smoking (OR = 2.91) and secondhand smoke exposure (OR = 2.35). Rozi et al. [20] also reported significantly higher risks of LBW and preterm birth among exposed mothers.

Chelchowska et al. [15] demonstrated a significant reduction in birth weight (approximately 230 g) and head circumference among infants of smoking mothers. Ein-Mor et al. [16], using urinary cotinine as a biomarker of exposure, reported reduced birth weight and head circumference in exposed neonates. Talati et al. [21] reported an average reduction in birth weight of approximately 317 g among offspring of mothers who smoked daily.

Ashford et al. [13] found that smoking cessation during pregnancy was associated with higher birth weight compared to continued smoking, supporting a dose-response relationship. In contrast, Ali et al. [12], evaluating preconception smokeless tobacco use, did not observe a significant association with low birth weight or other adverse birth outcomes.

Developmental and Neurocognitive Outcomes

**Figure 2:**
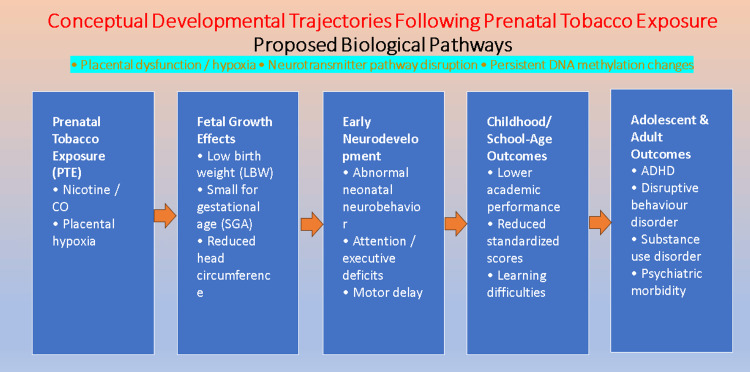
Conceptual framework illustrating developmental trajectories and biological pathways associated with prenatal tobacco exposure Prenatal tobacco exposure can cause placental hypoxia and impaired fetal growth, including low birth weight and reduced head circumference. These effects may lead to neurodevelopmental deficits, poor academic performance, and an increased risk of behavioral and psychiatric disorders later in life. ADHD: attention-deficit/hyperactivity disorder

Seven studies assessed developmental, cognitive, academic, or psychiatric outcomes (Table 3). Prenatal tobacco exposure was associated with impaired neurobehavior and cognitive performance in infancy and childhood (Figure 2). Ashford et al. [13] reported abnormal neonatal neurobehavior among exposed infants. Fuemmeler et al. [17] and Moore et al. [19] observed reduced attention, memory, executive function, and fine motor skills among exposed children.

**Table 3:** Developmental outcomes associated with prenatal tobacco exposure ADHD: attention-deficit/hyperactivity disorder; TS: Tourette syndrome; NIH: National Institutes of Health; ASQ: ages and stages questionnaire; OR: odds ratio; β: beta coefficient. aHR: adjusted hazard ratio

Study	Exposure	Developmental outcome	Measurement	Effect estimate	Statistical significance
Ashford et al. [13]	Prenatal	Neonatal neurobehavior	Biomarker	Abnormal neurobehavior	p<0.05
Fuemmeler et al. [17]	Prenatal	Cognition, attention	NIH toolbox	β=-0.58 to -1.29	p<0.05
Browne et al. [14]	Prenatal	ADHD, TS	Registry	aHR=1.66	95% CI: 1.17-2.35
Kristjansson et al. [18]	Prenatal	Academic scores	National tests	β=-2.91 to -6.43	p<0.001
Moore et al. [19]	Prenatal	Motor skills	ASQ-3	OR=3.9	p<0.05
Talati et al. [21]	Prenatal	Adult psychiatric outcomes	Clinical interview	OR=2.66	p<0.05

Long-term academic outcomes were examined by Kristjansson et al. [18], who reported significantly lower standardized academic scores among children prenatally exposed to tobacco. Psychiatric outcomes were evaluated in longitudinal cohorts. Browne et al. [14] reported increased risks of Tourette syndrome, chronic tic disorder, and attention-deficit/hyperactivity disorder in exposed offspring. Talati et al. [21] demonstrated elevated risks of disruptive behavior disorder and substance use disorder in adulthood among individuals exposed prenatally to maternal smoking.

Risk of Bias Assessment

Using the ROBINS-I tool [10], most studies were rated as having low-to-moderate or moderate overall risk of bias. Serious risk of bias was identified in three studies (Agena et al. [11], Rozi et al. [20], and Talati et al. [21]), primarily due to potential residual confounding and incomplete adjustment for socioeconomic and behavioral factors. Selection bias and outcome measurement bias were generally rated as low across registry-based and biomarker-validated studies. Detailed domain-wise assessments are presented in Table 4.

**Table 4:** Risk of bias assessment using ROBINS-I ROBINS-I: risk of bias in non-randomized studies of interventions

Study	Confounding	Selection	Exposure classification	Missing data	Outcome measurement	Overall risk
Agena et al. [11]	Serious	Low	Moderate	Low	Low	Serious
Aziz Ali et al. [12]	Moderate	Low	Moderate	Moderate	Low	Moderate
Ashford et al. [13]	Moderate	Low	Moderate	Low	Low	Moderate
Browne et al. [14]	Moderate	Low	Low	Low	Low	Low-moderate
Chelchowska et al. [15]	Moderate	Low	Low	Low	Low	Low-moderate
Ein-Mor et al. [16]	Moderate	Low	Low	Low	Low	Low
Rozi et al. [20]	Serious	Moderate	Moderate	Moderate	Low	Serious
Talati et al. [21]	Serious	Low	Moderate	Moderate	Moderate	Serious
Wang et al. [22]	Moderate	Low	Low	Low	Low	Moderate

Summary of Effect Estimates

Across included studies reporting categorical low birth weight (LBW), adjusted odds ratios ranged from 2.24 to 2.91 [11,20,22]. In addition, several studies reported significant reductions in continuous birth weight measures ranging from approximately 230 g to 317 g among exposed infants [15,21]. Reported reductions in continuous birth weight ranged from approximately 230 g to 317 g among exposed infants [15,21]. Cognitive and academic outcomes demonstrated negative beta coefficients ranging from -0.58 to -6.43 [17,18]. Psychiatric outcomes showed increased risks, with odds ratios up to 2.66 for disruptive behavior disorder [21]. A consolidated overview of effect estimates is presented in Table 5.

**Table 5:** Summary of effect estimates AOR: adjusted odds ratio; OR: odds ratio; CI: confidence interval; NR: not reported; DBD: disruptive behavior disorder; FHS: firsthand smoking; LBW: low birth weight; BW: birth weight.

Study	Exposure	Outcome	Effect estimate	95% CI	Sample size
Agena et al. [11]	Smoking	LBW	AOR=2.31	NR	381
Chelchowska et al. [15]	Smoking	BW	β=-230 g	-361.5 to -212.5	150
Talati et al. [21]	≥10 cig/day	DBD	OR=2.66	1.15-6.16	238
Wang et al. [22]	FHS	LBW	OR=2.91	1.49-5.68	~18000

Overall, the findings indicate consistent associations between prenatal tobacco exposure and reduced fetal growth, impaired neurocognitive development, poorer academic performance, and increased psychiatric morbidity. Evidence for smokeless tobacco exposure remains limited and inconclusive [12].

Certainty of Evidence

Using the GRADE approach, the certainty of evidence for categorical low birth weight outcomes was rated as moderate, supported by consistent findings across multiple observational studies. Evidence for broader fetal growth measures, including continuous birth weight reduction and head circumference, was also rated as moderate. Neurocognitive and academic outcomes were graded as moderate certainty. Evidence for preterm birth, psychiatric outcomes, and smokeless tobacco exposure was rated as low certainty due to fewer studies, heterogeneity in outcome assessment, and imprecision (Table 6).

**Table 6:** GRADE evidence profile GRADE: Grading of Recommendations Assessment, Development and Evaluation

Outcome	No. of studies	Study design	Risk of bias	Overall certainty
Low birth weight	7	Observational	Moderate	Moderate
Fetal growth	6	Observational	Moderate	Moderate
Preterm birth	4	Observational	Moderate	Low
Neurocognitive outcomes	3	Longitudinal	Moderate	Moderate
Academic performance	1	Registry	Low	Moderate
Psychiatric outcomes	2	Longitudinal	Moderate-serious	Low
Smokeless tobacco outcomes	1	Secondary analysis	Moderate	Low

Discussion

This review synthesizes evidence across multiple domains, including birth outcomes, neurodevelopment, and long-term psychiatric effects. This systematic review synthesizes findings from 12 observational studies examining the association between prenatal tobacco exposure and birth and developmental outcomes. Overall, the evidence demonstrates consistent associations between maternal smoking during pregnancy and reduced fetal growth, impaired neurodevelopment, poorer academic performance, and increased psychiatric morbidity. These findings are consistent with established epidemiological literature and biological models of fetal nicotine exposure [3,4,23,24]. These findings are consistent with prior meta-analyses demonstrating increased risks of low birth weight and preterm birth among smoking mothers [4,25]. However, the present review extends previous work by integrating recent longitudinal evidence on neurodevelopmental and psychiatric trajectories.

Birth Outcomes

The present review confirms a strong association between prenatal tobacco exposure and low birth weight. Agena et al. [11], Wang et al. [22], and Rozi et al. [20] reported significantly increased odds of low birth weight among exposed infants. Chelchowska et al. [15] and Ein-Mor et al. [16] demonstrated reductions in birth weight and head circumference, including studies incorporating biomarker-based exposure validation. Talati et al. [21] further reported clinically meaningful reductions in birth weight among offspring of daily smokers.

These findings are consistent with prior evidence indicating that nicotine and carbon monoxide impair placental perfusion and oxygen delivery, contributing to intrauterine growth restriction [4,24]. The observation that smoking cessation during pregnancy was associated with improved birth weight in Ashford et al. [13] supports a dose-response relationship. In contrast, Ali et al. [12] did not observe significant associations between preconception smokeless tobacco use and adverse birth outcomes. Differences in exposure timing, nicotine content, and potential underreporting may explain this discrepancy.

Neurodevelopmental and Cognitive Outcomes

Several studies identified associations between prenatal tobacco exposure and impaired neurocognitive performance. Ashford et al. [13] reported abnormal neonatal neurobehavior, while Fuemmeler et al. [17] and Moore et al. [19] observed deficits in attention, memory, executive function, and fine motor skills. These findings are biologically plausible, as nicotine disrupts neuronal migration and synaptic formation during critical periods of brain development [3,7].

Longitudinal evidence from Kristjansson et al. [18] demonstrated lower standardized academic scores among exposed children, suggesting that early neurodevelopmental differences may persist into school age. However, the magnitude of cognitive effects varied across studies, and deficits were often domain-specific rather than global, indicating that higher-order executive processes may be particularly vulnerable.

Beyond direct toxic and vascular effects, emerging evidence suggests that epigenetic modifications may represent a key biological mechanism linking prenatal tobacco exposure to long-term neurodevelopmental and psychiatric outcomes. Genome-wide DNA methylation studies have demonstrated differential methylation patterns in cord blood and neonatal tissues among offspring exposed to maternal smoking during pregnancy [7]. Notably, methylation changes have been identified in genes involved in neurodevelopmental regulation, synaptic signaling, and dopaminergic pathways. Richmond et al. [8] further reported that some smoking-associated DNA methylation signatures persist from birth into adolescence and adulthood, indicating long-lasting molecular imprinting. These epigenetic alterations may influence gene expression involved in neuronal differentiation, impulse control, and behavioral regulation, thereby increasing vulnerability to attention-deficit/hyperactivity disorder, disruptive behavior disorders, and other psychiatric conditions observed in exposed cohorts [7,8]. The persistence of these methylation changes across the life course provides biological plausibility for the observed associations between in utero tobacco exposure and later psychiatric morbidity. Although causality cannot be definitively established, the convergence of epidemiological findings with molecular epigenetic evidence strengthens the hypothesis that prenatal tobacco exposure exerts durable neurobiological effects extending beyond birth outcomes. Notably, differential methylation has been observed in genes such as AHRR and CYP1A1, which are involved in xenobiotic metabolism and have been proposed as biomarkers of prenatal smoke exposure [7]. Persistent methylation signatures may influence stress-response systems and executive function regulation, potentially mediating long-term vulnerability to behavioral and psychiatric disorders [7,8].

Epigenetic and Biological Mechanisms

Recent studies have further demonstrated that DNA methylation signatures in placental and neonatal tissues can serve as biomarkers of prenatal tobacco exposure and are associated with long-term developmental and health outcomes [26-28]. These alterations may contribute to long-term deficits in attention, executive function, and behavioral regulation. In addition, emerging epigenetic research has shown that prenatal tobacco exposure is associated with persistent DNA methylation changes in offspring, including genes involved in neurodevelopment and immune regulation. Such epigenetic modifications may serve as molecular mediators linking in utero exposure to later neurodevelopmental and psychiatric outcomes, thereby providing further support for a developmental origins framework of disease [29,30].

Psychiatric and Behavioral Outcomes

Associations between prenatal tobacco exposure and later psychiatric morbidity were reported by Browne et al. [14] and Talati et al. [21]. Increased risks of Tourette syndrome, attention-deficit/hyperactivity disorder, disruptive behavior disorder, and substance use disorder were observed among exposed offspring. Although these findings align with neurobiological hypotheses involving dopaminergic and serotonergic pathway disruption, psychiatric outcomes are susceptible to residual confounding by familial and environmental factors. Despite multivariable adjustments, complete control for parental psychopathology and postnatal influences remains challenging.

Smokeless Tobacco Exposure

Evidence regarding smokeless tobacco exposure was limited. The study by Ali et al. [12] did not demonstrate significant associations with adverse birth outcomes. Given the limited number of eligible studies and variability in smokeless tobacco products, definitive conclusions cannot be drawn. Further prospective studies are needed to clarify these associations.

Risk of Bias and Methodological Considerations

Most included studies were rated as having moderate risk of bias using the ROBINS-I tool [10], primarily due to potential residual confounding and reliance on self-reported exposure. Studies incorporating biomarker validation [16,17] and registry-based outcomes [14,18] strengthened internal validity. Although observational design limits causal inference, the consistency of findings, presence of dose-response relationships [13,22], and biological plausibility [3,24] support a likely association between prenatal tobacco exposure and adverse outcomes. The presence of dose-response relationships strengthens causal inference under Hill’s criteria, particularly when combined with biological plausibility and consistency across populations.

Implications for Public Health and Clinical Practice

The findings reinforce the importance of tobacco cessation interventions during preconception and pregnancy. Reduced fetal growth and long-term neurodevelopmental and psychiatric effects have implications for healthcare utilization and educational outcomes. Evidence-based cessation programs incorporating behavioral counseling and appropriate pharmacotherapy may reduce prenatal exposure [5,23]. Routine screening for tobacco use in antenatal care remains essential. From a public health perspective, the long-term cognitive and psychiatric sequelae associated with prenatal tobacco exposure may contribute to cumulative educational disadvantage, healthcare burden, and intergenerational health inequities. Preventive strategies during preconception and pregnancy therefore have implications extending beyond immediate neonatal outcomes.

Limitations

This review has important limitations. Several included studies assessed prenatal tobacco exposure using maternal self-report, which is prone to recall and social desirability bias and may have resulted in exposure misclassification; only some studies used biomarker validation (e.g., cotinine). In addition, because the included evidence was observational, the findings represent associations and cannot establish definitive causality, and residual confounding (e.g., socioeconomic factors, parental mental health, co-substance use, and postnatal smoke exposure) may persist despite adjustments. Variability in exposure definitions (active, secondhand, smokeless tobacco) and outcome measurements also introduced heterogeneity and limited direct comparability across studies, while evidence for smokeless tobacco exposure remained limited.

Future Research

Future research should prioritize large prospective cohorts with repeated biomarker measurements, standardized developmental assessments, and extended follow-up into adulthood. Additional investigation into smokeless tobacco products and emerging nicotine delivery systems is warranted. Integration of genetic and epigenetic analyses may further clarify underlying mechanisms [7,8].

Ethics Statement

This study was conducted as a systematic review of previously published literature and did not involve human participants or animal subjects; therefore, institutional ethical approval was not required. The review protocol was prospectively registered with the International Prospective Register of Systematic Reviews (PROSPERO; registration number CRD42024555533) to ensure methodological transparency and reduce the risk of selective reporting.

Conflict of Interest Disclosures

The authors declare no conflicts of interest related to this study. The systematic review protocol was prospectively registered with PROSPERO (CRD42024555533), and all methods were conducted in accordance with the registered protocol.

## Conclusions

This systematic review indicates consistent associations between prenatal tobacco exposure and adverse birth outcomes, including reduced birth weight and impaired fetal growth, as well as subsequent neurodevelopmental, academic, and psychiatric challenges in offspring. Evidence from multiple observational studies supports a dose-response relationship and biological plausibility through mechanisms such as placental dysfunction, fetal hypoxia, and neurodevelopmental disruption. However, because the available evidence is predominantly observational, the findings should be interpreted as associations rather than definitive causal effects. Evidence regarding smokeless tobacco exposure remains limited and inconclusive. Given that prenatal tobacco exposure is preventable, strengthening tobacco cessation strategies during preconception and antenatal care remains a critical public health priority to improve maternal and child health outcomes.

## Appendices

The Consequences of Prenatal Tobacco Exposure: A Systematic Review of Birth Outcomes and Developmental Trajectories

PICO (PECO) Framework for Study Eligibility Criteria

Component

Description

Population (P)

Pregnant women and their offspring, irrespective of geographic location, socioeconomic status, or healthcare setting.

Exposure (E/I)

Maternal tobacco use during pregnancy, including active cigarette smoking and smokeless tobacco exposure (self-reported or biomarker-validated).

Comparator (C)

Pregnant women with no reported tobacco exposure during pregnancy.

Primary Outcomes (O1)

Low birth weight (LBW), preterm birth (PTB), small for gestational age (SGA), stillbirth, and miscarriage.

Secondary Outcomes (O2)

Neurodevelopmental and long-term health outcomes in offspring, including cognitive impairment, language delay, attention-deficit/hyperactivity disorder (ADHD), behavioral disorders, motor delay, emotional disturbances, respiratory morbidity, growth abnormalities, and chronic conditions.

Study Design (S)

Observational studies including cohort, case-control, and cross-sectional studies reporting quantitative associations.

PubMed

("Prenatal Exposure" OR "Maternal Smoking" OR "Tobacco Use During Pregnancy" OR "Cigarette Smoking in Pregnancy") AND ("Birth Weight" OR "Preterm Birth" OR "Low Birth Weight" OR "Small for Gestational Age" OR "Neonatal Outcomes" OR "Child Development" OR "Cognitive Development" OR "Behavioural Outcomes" OR "Neurodevelopment")

Filters Used

Filters applied: Full text, Clinical Study, Clinical Trial, Clinical Trial Protocol, Clinical Trial, Phase I, Clinical Trial, Phase II, Clinical Trial, Phase III, Clinical Trial, Phase IV, Controlled Clinical Trial, Observational Study, Pragmatic Clinical Trial, Randomized Controlled Trial, in the last 10 years, Humans, English, Female. 

Cochrane Library via Wiley

("regenerative endodontics" OR "regenerative endodontic therapy") AND ("traditional root canal therapy" OR "apexification") AND ("clinical outcomes" OR "success rates")

Google Scholar

"prenatal exposure" "maternal smoking" "tobacco use during pregnancy" "cigarette smoking in pregnancy" "birth weight" "preterm birth" "low birth weight" "small for gestational age" "neonatal outcomes" "child development" "cognitive development" "behavioral outcomes" "neurodevelopment"

Dimensions

("Type 2 Diabetes Mellitus" OR "T2DM" OR "Diabetes Mellitus, Type 2") AND ("Dental Implant" OR "Oral Implant" OR "Osseointegrated Implant") AND ("Microbiome" OR "Microflora" OR "Microbial Community" OR "Oral Microbiota")

Science Direct

("regenerative endodontics" OR "regenerative endodontic therapy") AND ("root canal therapy" OR "apexification") AND ("treatment outcome" OR "success rates"

PsycINFO

("prenatal exposure" OR "maternal smoking" OR "tobacco use during pregnancy" OR "cigarette smoking in pregnancy") AND ("birth weight" OR "preterm birth" OR "low birth weight" OR "small for gestational age" OR "neonatal outcomes" OR "child development" OR "cognitive development" OR "behavioral outcomes" OR "neurodevelopment")

Cochrane Library

"prenatal exposure" OR "maternal smoking" OR "tobacco use during pregnancy" OR "cigarette smoking in pregnancy"

Dimensions

("prenatal tobacco exposure" OR "maternal smoking" OR "nicotine exposure")

AND ("birth outcomes" OR "developmental trajectories" OR "cognitive development" OR "neurobehavioral outcomes")

Scopus

Search conducted in Title, Abstract, and Keywords:

TITLE-ABS-KEY ("prenatal tobacco exposure" OR "maternal smoking" OR

"cigarette smoking during pregnancy" OR "smokeless tobacco")

AND

TITLE-ABS-KEY ("low birth weight" OR LBW OR "preterm birth" OR PTB OR

"small for gestational age" OR SGA OR stillbirth OR miscarriage OR

neurodevelopment OR ADHD OR "behavioral disorder" OR "cognitive impairment")

AND PUBYEAR > 2013 AND PUBYEAR < 2026

## References

[1] World Health Organization (2013). WHO Recommendations for the Prevention and Management of Tobacco Use and Second-Hand Smoke Exposure in Pregnancy. Geneva: World Health Organization.

[2] Lange S, Probst C, Rehm J, Popova S (2018). National, regional, and global prevalence of smoking during pregnancy in the general population: a systematic review and meta-analysis. Lancet Glob Health.

[3] Slotkin TA (1998). Fetal nicotine or cocaine exposure: which one is worse?. J Pharmacol Exp Ther.

[4] Salmasi G, Grady R, Jones J, McDonald SD (2010). Environmental tobacco smoke exposure and perinatal outcomes: a systematic review and meta-analyses. Acta Obstet Gynecol Scand.

[5] U.S. Department of Health and Human Services (2014). The Health Consequences of Smoking—50 Years of Progress: A Report of the Surgeon General. Atlanta, GA: Centers for Disease Control and Prevention.

[6] DiFranza JR, Aligne CA, Weitzman M (2004). Prenatal and postnatal environmental tobacco smoke exposure and children's health. Pediatrics.

[7] Joubert BR, Håberg SE, Nilsen RM (2012). 450K epigenome-wide scan identifies differential DNA methylation in newborns related to maternal smoking during pregnancy. Environ Health Perspect.

[8] Richmond RC, Simpkin AJ, Woodward G (2015). Prenatal exposure to maternal smoking and offspring DNA methylation across the lifecourse: findings from the Avon Longitudinal Study of Parents and Children (ALSPAC). Hum Mol Genet.

[9] Page MJ, Moher D, Bossuyt PM (2021). PRISMA 2020 explanation and elaboration: updated guidance and exemplars for reporting systematic reviews. BMJ.

[10] Sterne JA, Hernán MA, Reeves BC (2016). ROBINS-I: a tool for assessing risk of bias in non-randomised studies of interventions. BMJ.

[11] Agena AG, Modiba LM (2019). Maternal and foetal medical conditions during pregnancy as determinants of intrapartum stillbirth in public health facilities of Addis Ababa: a case-control study. Pan Afr Med J.

[12] Aziz Ali S, Khan U, Abrejo F (2021). Use of smokeless tobacco before conception and its relationship with maternal and fetal outcomes of pregnancy in Thatta, Pakistan: findings from women first study. Nicotine Tob Res.

[13] Ashford K, McCubbin A, Barnett J, Blair LM, Lei F, Bush H, Breland A (2021). Longitudinal examination of prenatal tobacco switching behaviors and birth outcomes, including electronic nicotine delivery system (ENDS) and dual use. Matern Child Health J.

[14] Browne HA, Modabbernia A, Buxbaum JD (2016). Prenatal maternal smoking and increased risk for Tourette syndrome and chronic tic disorders. J Am Acad Child Adolesc Psychiatry.

[15] Chełchowska M, Gajewska J, Mazur J, Ambroszkiewicz J, Maciejewski TM, Leibschang J (2016). Serum pregnancy-associated plasma protein A levels in the first, second and third trimester of pregnancy: relation to newborn anthropometric parameters and maternal tobacco smoking. Arch Med Sci.

[16] Ein-Mor E, Berman T, Barnett-Itzhaki Z (2019). Newborn infant urinary cotinine and birth outcomes in the Jerusalem Environment Mother and Child Cohort Study. Int J Hyg Environ Health.

[17] Fuemmeler BF, Glasgow TE, Schechter JC (2023). Prenatal and childhood smoke exposure associations with cognition, language, and attention-deficit/hyperactivity disorder. J Pediatr.

[18] Kristjansson AL, Thomas S, Lilly CL, Thorisdottir IE, Allegrante JP, Sigfusdottir ID (2018). Maternal smoking during pregnancy and academic achievement of offspring over time: a registry data-based cohort study. Prev Med.

[19] Moore BF, Shapiro AL, Wilkening G (2020). Prenatal exposure to tobacco and offspring neurocognitive development in the healthy start study. J Pediatr.

[20] Rozi S, Butt ZA, Zahid N, Wasim S, Shafique K (2016). Association of tobacco use and other determinants with pregnancy outcomes: a multicentre hospital-based case-control study in Karachi, Pakistan. BMJ Open.

[21] Talati A, Wickramaratne PJ, Wesselhoeft R, Weissman MM (2017). Prenatal tobacco exposure, birthweight, and offspring psychopathology. Psychiatry Res.

[22] Wang X, Gao X, Chen D (2024). The effect of active and passive smoking during pregnancy on birth outcomes: a cohort study in Shanghai. Tob Induc Dis.

[23] Benowitz N, Dempsey D (2004). Pharmacotherapy for smoking cessation during pregnancy. Nicotine Tob Res.

[24] Zdravkovic T, Genbacev O, McMaster MT, Fisher SJ (2005). The adverse effects of maternal smoking on the human placenta: a review. Placenta.

[25] Dietz PM, England LJ, Shapiro-Mendoza CK, Tong VT, Farr SL, Callaghan WM (2010). Infant morbidity and mortality attributable to prenatal smoking in the U.S.. Am J Prev Med.

[26] Deng WQ, Cawte N, Campbell N (2024). Maternal smoking DNA methylation risk score associated with health outcomes in offspring of European and South Asian ancestry. Elife.

[27] Shorey-Kendrick LE, Davis B, Gao L (2024). Development and validation of a novel placental DNA methylation biomarker of maternal smoking during pregnancy in the ECHO program. Environ Health Perspect.

[28] Gould TJ (2023). Epigenetic and long-term effects of nicotine on biology, behavior, and health. Pharmacol Res.

[29] Castro EM, Lotfipour S, Leslie FM (2023). Nicotine on the developing brain. Pharmacol Res.

[30] Gittens OE, Folger AT, Zhang X, Ding L, Parikh NA, Mahabee-Gittens EM (2025). Examination of DNA methylation patterns in children born premature with prenatal tobacco smoke exposure. Toxics.

